# Tonic 5nM DA Stabilizes Neuronal Output by Enabling Bidirectional Activity-Dependent Regulation of the Hyperpolarization Activated Current via PKA and Calcineurin

**DOI:** 10.1371/journal.pone.0117965

**Published:** 2015-02-18

**Authors:** Wulf-Dieter C. Krenz, Edmund W. Rodgers, Deborah J. Baro

**Affiliations:** Department of Biology, Georgia State University, Atlanta, Georgia, United States of America; University of Missouri, UNITED STATES

## Abstract

Volume transmission results in phasic and tonic modulatory signals. The actions of tonic dopamine (DA) at type 1 DA receptors (D1Rs) are largely undefined. Here we show that tonic 5nM DA acts at D1Rs to stabilize neuronal output over minutes by enabling activity-dependent regulation of the hyperpolarization activated current (I _h_). In the presence but not absence of 5nM DA, I _h_ maximal conductance (G _max_) was adjusted according to changes in slow wave activity in order to maintain spike timing. Our study on the lateral pyloric neuron (LP), which undergoes rhythmic oscillations in membrane potential with depolarized plateaus, demonstrated that incremental, bi-directional changes in plateau duration produced corresponding alterations in LP I _h_G _max_ when preparations were superfused with saline containing 5nM DA. However, when preparations were superfused with saline alone there was no linear correlation between LP I _h_G_max_ and duty cycle. Thus, tonic nM DA modulated the capacity for activity to modulate LP I _h_ G _max_; this exemplifies metamodulation (modulation of modulation). Pretreatment with the Ca2+-chelator, BAPTA, or the specific PKA inhibitor, PKI, prevented all changes in LP I _h_ in 5nM DA. Calcineurin inhibitors blocked activity-dependent changes enabled by DA and revealed a PKA-mediated, activity-independent enhancement of LP I _h_G _max_. These data suggested that tonic 5nM DA produced two simultaneous, PKA-dependent effects: a direct increase in LP I _h_ G _max_ and a priming event that permitted calcineurin regulation of LP I _h_. The latter produced graded reductions in LP I _h_G _max_ with increasing duty cycles. We also demonstrated that this metamodulation preserved the timing of LP’s first spike when network output was perturbed with bath-applied 4AP. In sum, 5nM DA permits slow wave activity to provide feedback that maintains spike timing, suggesting that one function of low-level, tonic modulation is to stabilize specific features of a dynamic output.

## Introduction

Homeostatic mechanisms stabilize neuronal firing patterns [1]. Hyperpolarization activated, cyclic nucleotide gated (HCN) channels that mediate I_h_ are often the target of such mechanisms because of their wide-ranging influence on synaptic integration and neuronal excitability [2–5]. A number of homeostatic mechanisms in a variety of cell types produce transient or persistent alterations in I_h_ to maintain neuronal function [6–15]. Cognitive impairment associated with Fragile X Syndrome [16] and the loss of pacemaking in Parkinson’s disease [17] can be linked to improper homeostatic regulation of I_h_.

HCN channels influence several activity features that shape neuronal excitability and synaptic integration including membrane potential, firing threshold, resonance frequency, temporal summation and synaptic strength [5,18–20]. Each activity feature can be underpinned by the balance of two or more conductances [21]; for example, GABA_A_ receptors and HCN1 channels co-vary to preserve hippocampal neuron resting membrane potential [22], but in cortical pyramidal neurons, these two conductances vary inversely to sustain excitatory post synaptic potential summation [23]. KCNE2 and HCN channels may also be co-regulated to uphold ventrobasal and cortical layer 6 pyramidal neuron excitability [24]. In stomatogastric pyloric neurons, Kv4 channels that mediate the transient potassium current (I_A_) co-vary with HCN channels to maintain the timing of neuronal activity [14,15,25].

**Fig 1:**
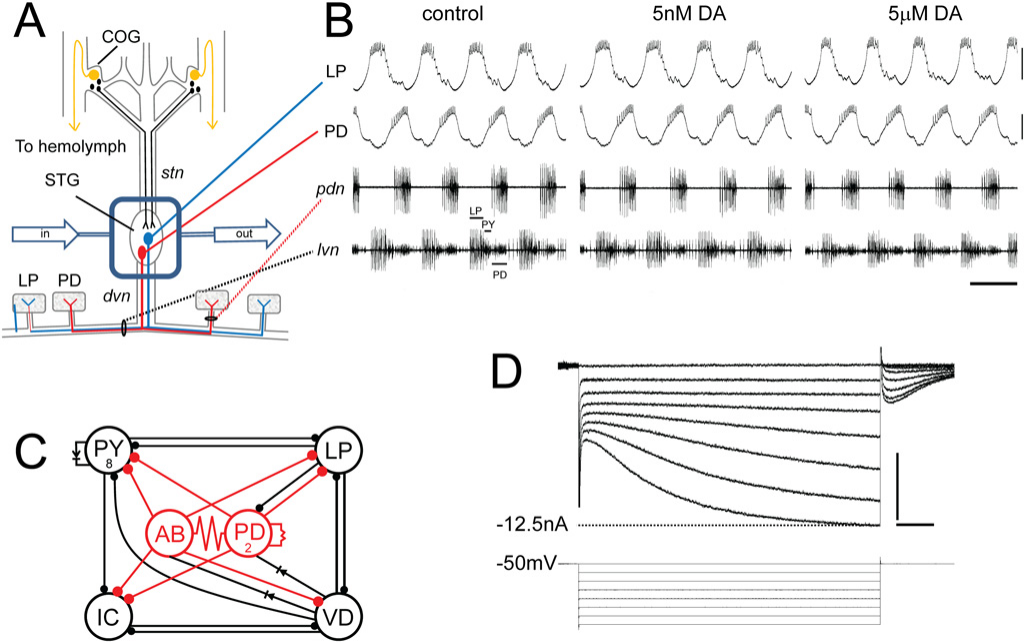
The experimental model. (**A) *In situ* preparation**. The STNS is dissected & pinned in a dish. The commissural ganglia (CoGs) contain DA neurons that project to the STG (black) and L-cells, which are the source of neurohormonal DA (gold). The well surrounding the STG (blue rectangle) is continuously superfused with saline (in/out arrows). There are ~30 neurons in the STG; 2 are drawn: pyloric dilator (PD), lateral pyloric (LP). Network neurons interact locally within the STG neuropil and can project axons to striated muscles surrounding the foregut. The diagram shows that PD & LP neurons project their axons through identified nerves to innervate muscles (rectangles). **(B) Spontaneous pyloric network output**. The top 2 traces are intra-cellular recordings from the *in situ* preparation diagrammed in A. The bottom 2 traces represent extra-cellular recordings from identified motor nerves containing pyloric neuron axons. The spikes from three pyloric neurons are indicated on *lvn*. These simultaneous recordings demonstrate the spontaneous, recurrent, rhythmic motor pattern produced by the circuit; scale bars, 10mV & 500ms. **(C) The pyloric circuit**. The pyloric network comprises 14 neurons. The diagram represents pyloric neuron interactions within the STG. Open circles represent the 6 cell types, numbers indicate more than 1 cell within a cell type: anterior burster (AB), inferior cardiac (IC), ventricular dilator (VD); pyloric constrictor (PY); filled circles, inhibitory chemical synapses; resistors & diodes, electrical coupling; red, pacemaker kernel and its output connections. **(D) Two electrode voltage clamp experiment**. Top: Typical LP I_h_ recording; Bottom: voltage protocol; scale bars, 500ms and 5nA.

The homeostatic mechanisms that preserve activity features by maintaining their underlying conductance correlations are largely undefined. We have been addressing their organization using an invertebrate model. The pyloric network generates a continuous rhythmic motor output within a limited frequency range (Fig. 1). The proper frequency range is preserved, in part, by the lateral pyloric neuron (LP) [26]. The timing of LP activity is critical for this function [27,28], and the ratio of LP I_A_:I_h_ is one of the factors that determines the timing of neuronal activity [29,30]. Population studies indicate that the timing of LP activity [29,31] and the LP I_A_:I_h_ ratio [32] are conserved across individuals. Moreover, if LP I_A_ is experimentally perturbed, then there is a compensatory alteration in LP I_h_ to preserve the timing of LP activity [14,15]. Together the data suggest that homeostatic mechanisms exist to sustain a positive correlation between LP I_A_ and I_h_ in order to maintain the timing of LP activity, and hence, an appropriate network output frequency. Tonic 5nM DA may enable such mechanisms.

In discussing DA-enabled homeostasis we employ the terms metaplasticity and metamodulation; therefore, we define these terms here. The prefix “meta” comes from the Greek preposition/prefix meaning beyond (among other things), and “meta” is often used to indicate a second order process. For example, metadata refers to data about data, metamodulation denotes modulation of modulation and metaplasticity means plasticity of plasticity. Here we broadly define modulation as a slow (seconds to minutes), stimulus-induced change in function that does not significantly outlast the stimulus, while plasticity is defined as a slow, stimulus-induced change in function that significantly outlasts the stimulus [33]. According to these definitions, many types of stimuli can produce both modulation and plasticity, including monoamines and neuronal activity. The same types of stimuli can also produce metamodulation and metaplasticity. Metamodulation and metaplasticity occur when one stimulus alters the ability of a second stimulus to produce a change in function or alters the modulation/ plasticity normally elicited by that second stimulus [34–41].

Low-level, tonic modulation may stabilize neuronal and circuit output by evoking metaplasticity and metamodulation. We previously demonstrated that tonic 5nM DA altered the capacity of activity to produce a persistent change in LP I_h_ G_max_ (metaplasticity) by enabling a mechanism that relied on the RNAi pathway and mTOR-dependent translation [42,43]. Interestingly, the same mechanism persistently regulated LP I_A_ G_max_ [43–45], suggesting that this mechanism may co-regulate these two conductances to preserve the LP I_A_:I_h_ ratio over the long-term. In this manuscript we demonstrate that tonic 5nM DA changes the capacity for activity to modulate I_h_ G_max_ (metamodulation). Together with previous work [15,42], the data presented here indicate that this metamodulation maintains the LP I_A_:I_h_ ratio and the timing of LP activity over the short-term, and they suggest a simple working model for the molecular mechanism involved.

## Materials and Methods

### Animals

California spiny lobsters, *Panulirus interruptus*, were purchased from Marinus Scientific (Long Beach, CA) and Catalina Offshore Products (San Diego, CA). Lobsters were maintained at 18°C in aerated and filtered seawater. Animals were anesthetized on ice before dissection.

### Chemicals and peptides

Tetrodotoxin (TTX), myristoylated PKI_(14–22)_ and calcineurin autoinhibitory peptide (CiP) were purchased from Tocris Bioscience (Bristol, UK). BAPTA-AM, ryanodine and xestospongin C were from Cayman Chemical (Ann Arbor, MI). All other chemicals were purchased from Sigma-Aldrich (St. Louis, MI). DA was made fresh every 30min to minimize oxidation. PKI is an effective blocker of the PKA catalytic subunit in crustaceans [43,46,47]. CiP and FK506 block calcineurin activity in crustacean neurons at the indicated dosages [48–50]. In the spiny lobster, BAPTA, ryanodine and xestospongin C disrupt Ca^2+^ dynamics at the indicated dosages by chelating Ca^2+^, blocking ryanodine receptor function and inhibiting IP_3_ receptor function, respectively [51].

### Experimental Preparation

The STNS was dissected and pinned in a Sylgard lined Petri dish using standard techniques [52]. The stomatogastric ganglion (STG) was desheathed and isolated with a Vaseline well. The STG was superfused with saline consisting of (in mM) 479 NaCl, 12.8 KCl, 13.7 CaCl_2_, 39 Na_2_SO_4_, 10 MgSO_4_, 2 glucose, 4.99 HEPES, 5 TES at pH 7.4. Extracellular recordings from the pyloric dilator nerve (*pdn)* and lateral ventricular nerve (*lvn)* were obtained with stainless steel pin electrodes and a differential AC amplifier (A-M Systems, Everett, WA) as previously described [53]. Intracellular somatic recordings were obtained using glass microelectrodes filled with 3M KCl (20–30 MΩ) and Axoclamp 2B or 900A amplifiers (Molecular Devices, Foster City, CA). Neurons were identified by correlating action potentials from somatic intracellular recordings with extracellularly recorded action potentials on identified motor nerves, and by their characteristic shape and timing of oscillations.

### Somatic two-electrode voltage clamp (TEVC)

For TEVC of LP I_h_, the LP neuron was impaled with two micropipettes (8–10 MΩ when filled with 3 M KCl) connected to Axoclamp 2B or 900A amplifiers (Molecular Devices, Foster City, CA). The well surrounding the STG was superfused with saline containing TTX for 5min. The LP was clamped to a-50mV holding potential using pClamp software. I_h_ was elicited using a series of 4s hyperpolarizing voltage steps, from-60mV to-120mV in 10mV increments with 6s between steps. Steady state peak currents were measured by fitting the current trace back to the beginning of the hyperpolarizing voltage step or by subtracting the initial fast leak current from the slowly developing peak of I_h_ at the end of each negative voltage step. Currents were converted to conductance (G = I_peak_/(V_m_-V_rev_) and fitted to a 1^st^ order Boltzmann equation. V_rev_ I_h_ = -35mV [54]. TEVC experiments were done at 19–22°C as measured with a probe in the bath. Temperature did not change by more than 1°C during any given experiment.

### Peptide injection

The calcineurin autoinhibitory peptide (CiP) was diluted in water to a working concentration of 50μg/ml and fast green was added to 0.04% to visualize injections. Microloaders (Eppendorf) were used to directly fill glass pipettes with the solution (8–15 MΩ when filled with 3M KCl). Because of the high resistance and viscosity of the peptide solution, pipette tips were broken before injection by gently touching a Kim wipe. The peptide was pressure injected into LP neurons using a Picospritzer III (General Valve/Parker Hannifin). Only two pressure pulses (on average 28psi and 30ms) separated by 30s were applied. Intracellular recording during the injection showed that the injection procedure had no effect on the LP voltage envelope and firing properties. Extracellular recordings were used to continuously monitor the activity of the LP neuron before, during and for 10–20min after peptide injection.

### Statistical analyses

The data were checked for normality and analyzed using parametric statistics. In the one case where data were not normally distributed, non-parametric tests were used. All data were analyzed using Prism Statistical software package (Graphpad). Significance threshold was set at p<0.05 in all cases. Statistical outliers were excluded if their values fell outside the range delimited by the mean±(2×stdev). Means and standard errors are presented unless otherwise noted. ANOVAs were usually followed by either Dunnett’s post-hoc tests that compare all columns to a single column or Tukey’s post-hoc tests that make all pairwise comparisons.

## Results

### The experimental model

Our model system is the crustacean STNS (Fig. 1A), which contains several pattern generators that control the movements of the crustacean foregut [55]. The 14-neuron pyloric pattern generator, located in the stomatogastric ganglion (STG), is a recurrent inhibitory circuit that generates a continuous rhythmic motor pattern *in vivo* [56] and *in situ* (Fig. 1B). Pyloric network output is tempered by over 30 modulators, including DA [55]. DA projection neurons in the commissural ganglia (COGs) use volume transmission to release DA into the STG neuropil (Fig. 1A) [57]. Thus, targets of DA projection neurons experience two types of transmission: a transient increase in DA (~μM) near projection neuron release sites and tonic exposure to ~nM DA [58,59]. In addition, the STG is exposed to neurohormonal DA [55]. Bath application of μM but not nM DA produces immediate and reversible alterations in circuit output, including an increase in cycle frequency, a decrease in LP burst duration, an LP phase advance and an increase in LP intraburst spike frequency (Fig. 1B). Studies show that μM DA generates these changes by altering pyloric neuron synaptic strengths, Ca^2+^ dynamics and the biophysical properties of their ion channels [30,36,51,60–75]. On the other hand, little is known about the functions of tonic nM DA. Previous work showed that tonic nM DA could regulate pyloric neuron peripheral spiking and improves the temporal fidelity of axonal spike propagation [76–78]. In order to further examine the function of tonic nM DA, we performed experiments wherein the STNS was dissected out, pinned in a dish and constantly superfused to prevent the accumulation of tonic modulators; then, 5nM DA was added back to the superfusate (Fig. 1A). In this manuscript we examined the effects of tonic DA on LP I_h_, where LP I_h_ was measured with a standard TEVC protocol (Fig. 1D). LP expresses type 1 DA receptors (D1Rs) but not type 2 [47]. STNS DA receptors can act through canonical pathways [79] and behave like their mammalian counterparts when expressed in human cell lines [80,81].

### Tonic 5nM DA stabilizes LP activity phase

A previous study from our lab suggested that bath applied 5μM DA acted at low and high affinity receptors to simultaneously alter and stabilize network output, respectively [15]. The pyloric network operates within a limited range of cycle frequencies. Multiple network interactions maintain output within this optimal range; for example, the LP slows increasing network cycle frequencies by inhibiting the pacemaker kernel (Fig. 1C) [26]. The timing of LP inhibition onto the pacemaker is critical for this function [27]. Timing is regulated, in part, by the ratio of LP I_A_:I_h_ [30]. Bath application of 5μM DA affected both conductances: It reduced LP I_A_ G_max_ [47], and it endowed LP I_h_ with activity dependence [15]. As a result, the reduction in I_A_ produced changes in activity that drove a compensatory decrease in I_h_, which in turn, maintained the LP I_A_:I_h_ ratio and the timing of LP activity. The threshold concentrations of DA required for altering the properties of LP I_A_ and I_h_ were μM and nM, respectively. This work suggested that the role of tonic nM DA may be to stabilize network function by maintaining conductance correlations over the short term. To further test this hypothesis here, changes in circuit output were examined in the presence and absence of DA while superfusing the preparation with 1mM 4-AP, an I_A_ blocker that was previously shown to significantly alter network activity at this concentration [82].

**Fig 2:**
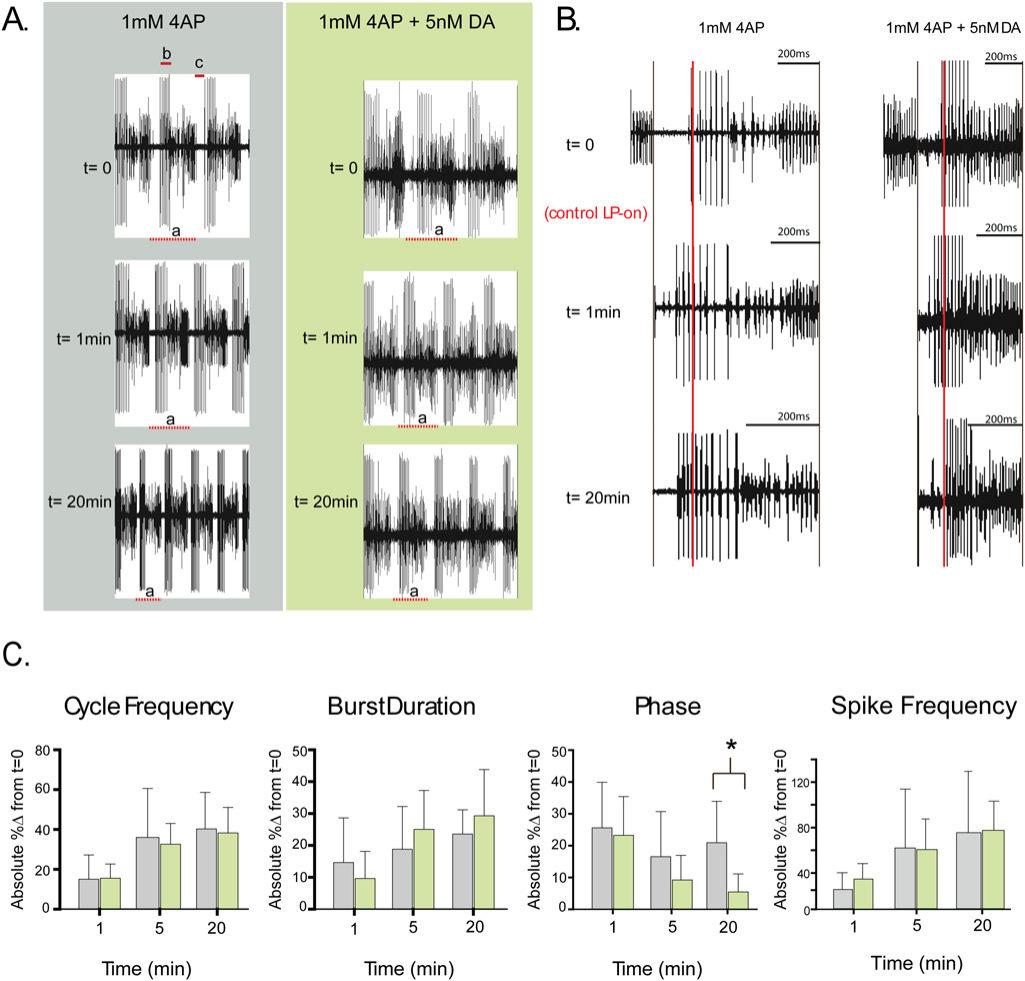
Tonic nM DA stabilizes the timing of LP activity over the short term. **(A) Pyloric output is altered by 1mM 4AP**. Representative experiments showing the motor output at three time points for a preparation continuously superfused with 1mM 4AP (blue) or 1mM 4AP + 5nM DA (green) beginning at t = 0. Each trace from one preparation represents the same length of time. Cycle period is measured as the last spike in a PD burst to the last spike in the subsequent PD burst and is indicated by “a” in each trace. LP burst duration is indicated by “b”, and LP-on delay, which is the time between the last PD spike and the first LP spike, is indicated by “c”. Intraburst spike frequency is the number of spikes per burst divided by burst duration. **(B) The timing of LP activity is stabilized by 5nM DA**. In order to visually compare the timing of LP activity at t = 0,1min and 20min, we performed the following manipulations on each the two sets of traces shown in panel A. First, we aligned one cycle period for each of the three time points (dashed lines below traces) by the last spike in the preceding PD burst. Second, the horizontal axes of the 1min and 20min traces were scaled to match the length of the t = 0 trace. The red line marks the point in the cycle when the first LP spike occurs for the t = 0 trace. Note that in both preparations a 1min 1mM 4AP bath application caused LP activity to occur earlier in the cycle. LP activity remained phase advanced only in the absence of 5nM DA. **(C) 5nM DA preserves the timing of LP activity phase but not cycle frequency, or LP burst duration and intraburst spike frequency**. The absolute value of the percent change in cycle period, LP burst duration, LP-on phase (c÷a) and LP intraburst spike frequency is plotted (mean+stdev) at 1min,5min and 20min for preparations superfused with 1mM 4AP (blue) or 1mM 4AP+5nM DA (green). Asterisks indicate significant differences between treatment groups as determined using 2-way ANOVAs with post-hoc Bonferroni’s multiple comparisons tests. **Cycle period**: Time, F(2,38) = 47.62, p<0.0001; Treatment, F(1,19) = 0.1388, p = 0.7136; Interaction F(2,38) = 0.3760, p = 0.6891. **LP burst duration**: Time, F(2,38) = 9.257, p = 0.0005; Treatment, F(1,19) = 0.4796, p = 0.4970; Interaction, F(2,38) = 1.876, p = 0.1672. **LP phase**: Time, F(2,36) = 10.46, p = 0. 0003; Treatment, F(1,18) = 4.659, p = 0.0446; interaction F(2,36) = 2.645, p = 0.0848. **LP intraburst spike frequency**: Time, F(2,36) = 51.99, p<0.0001; Treatment, F(1,18) = 1.291, p = 0.2708; Interaction F(2,36) = 1.387, p = 0.2627. Note that absolute values were chosen because in some cases a given parameter could increase or decrease over time according to the preparation, so averaging did not reflect the degree of change that was observed.

The STNS was dissected, pinned in a dish and continuously superfused with saline (Fig. 1A). Extracellular recordings from the *lvn* and *pdn* were used to record activity throughout the experiment. At t = 0, 5nM DA was or was not (control) added to the superfusate along with 1mM 4-AP to reduce peak I_A_ by ~20%. Previous work indicated that bath application of 5nM DA had no immediate effect on motor output relative to saline controls [42], and this can also be observed in Fig. 1B. In contrast, as previously reported [82], a 1min bath application of 1mM 4AP altered network cycle frequency and neuronal phasing, burst durations and intraburst spike frequencies in both DA-treated and control preparations (Fig. 2A-B). Measurements indicated that the changes in cycle frequency, LP burst duration and LP intraburst spike frequency persisted throughout the 4AP application for both treatment groups (Fig. 2C); on the other hand, LP-on phase (the point in the cycle where LP begins firing) recovered over minutes in the DA-treated but not control preparations (Fig. 2B), and by 20min the change in LP-on phase was significantly greater in control relative to DA-treated preparations (Fig. 2C). These data indicated that 5nM DA could stabilize the timing of LP activity phase when network output was altered.

Using dynamic clamp we previously ablated DA-induced changes in LP I_h_ G_max_ and showed that they were responsible for LP-on phase recovery in 5μM DA [15]. In order to determine if I_h_ was necessary for the DA-dependent stabilization of LP-on phase in 4AP, experiments in 4AP were repeated but 0.5mM CsCl was also added to the superfusate at t = 0 to block I_h_ by ~30% [42]. Under these conditions LP-on phase was not maintained in the DA treatment group. At t = 20min in 5nM DA+1mM 4AP+ 0.5mM CsCl, the mean absolute percent change in LP-on phase was 32±20, and there was no difference in the change in phase between preparations superfused with 4AP vs. 4AP+5nM DA+ 0.5mM CsCl (Two way ANOVA: Time, F(2,26) = 12.88, p = 0. 0001; Treatment, F(1,13) = 1.520, p = 0.2394; interaction F(2,26) = 1.995, p = 0.1563). In sum, the data suggest that when circuit output is altered by reducing I_A_, tonic nM DA enables an I_h_-dependent mechanism to stabilize LP activity phase. We next investigated the mechanism involved.

### Tonic 5nM DA-enables bi-directional activity-dependent regulation of LP I_h_

We previously observed a significant increase in LP I_h_ G_max_ after a 10min activity blockade in the presence but not absence of 5nM DA, and in neither case was the voltage dependence of LP I_h_ altered [15]. Furthermore, in the absence of activity blockade, tonic 5nM DA had no significant effect on LP I_h_ G_max_. Thus, tonic 5nM DA changed the capacity of the modulator (neuronal activity) to alter LP I_h_ G_max_. Here we extend those studies to better understand how tonic 5nM DA regulates the activity dependence of LP I_h_ G_max_.

A prominent feature of pyloric neurons is their slow wave voltage oscillations with depolarized plateaus (Fig. 1B). During the oscillation, LP membrane potential ranges from roughly-62mV to-42mV, and spikes ride on top of the depolarized plateau. The timing of the spikes depends upon the rising phase of the voltage oscillation, and the I_A_:I_h_ ratio regulates the slope of the rising phase, among other things [30]. Thus, it seemed reasonable to think that slow wave activity might somehow provide feedback to maintain the I_A_:I_h_ ratio and the timing of LP activity.

**Fig 3:**
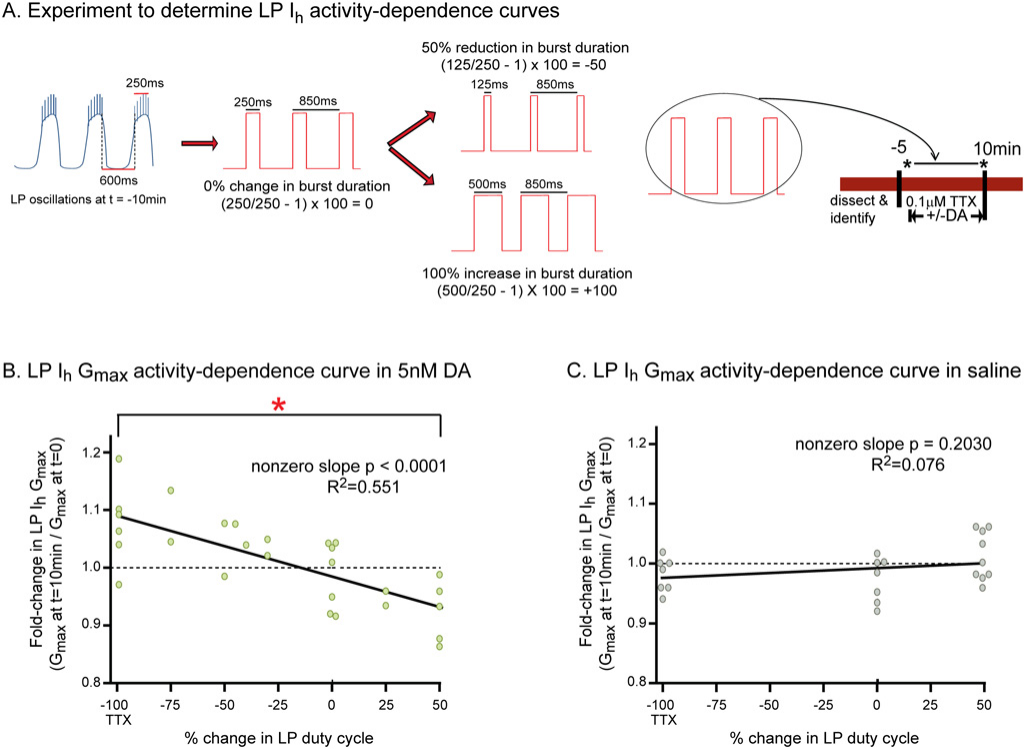
Tonic 5nM DA endows LP I_h_ with activity dependence. **(A) Experimental protocol**. Activity was recorded with extracellular electrodes throughout the experiment. Measures of activity were obtained at t = -10min. The diagram shows how these measures were used in conjunction with TEVC to create a recurring voltage step that mimicked slow oscillatory activity at t = -10min. A change in LP burst duration was created by varying the length of the depolarizing step, as shown. Note that cycle period was maintained regardless of the change in burst duration; i.e., a change in burst duration was accompanied by a corresponding change in interburst duration. Duty cycle is defined as burst duration/cycle period; thus, our manipulations altered burst duration and duty cycle to the same extent. At t = -5min TTX was added to the superfusate. At t = 0, LP I_h_ was measured with TEVC (black asterisk). Afterward, from t = 0 to 10min, 5nM DA was **(B)** or was not **(C)** added to the superfusate and TEVC was used to either hold the cell at its initial membrane potential in TTX (-100), or implement a recurrent voltage step (-75 to +50). At t = 10min, LP I_h_ was again measured with TEVC (black asterisk). The experiment terminated at this point and a single preparation was not used further for additional experiments. **(B-C) LP I**_**h**_ **activity dependence curves**. For every experiment in 5nM DA **(B)** or saline **(C)**, the fold-change in LP I_h_ G_max_ at t = 10min (i.e. G_max_ at t = 10÷G_max_ at t = 0) was plotted against the % change in LP duty cycle and a linear regression was used to fit the data. Each data point on the plots represents one experiment and 51 animals were used to obtain the data shown in the plots; -100 on the *x*-axis represents experiments in TTX without a recurring step, i.e., complete activity blockade. Each line represents a linear regression analysis and the resulting R^2^ and p values are shown on the graph. Red asterisk indicates that LP I_h_ G_max_ was significantly different at-100 and +50 in the presence but not absence of 5nM DA as determined using one-way ANOVAs with Tukey’s multiple comparisons post hoc tests to analyze the-100, 0, and +50 groups (**5nM DA**: F(2,17) = 8.464; p = 0.0035; **saline**: F(2,22) = 2.326; p = 0.1236).

We first examined if/how slow wave activity regulated LP I_h_ G_max_ in the presence and absence of tonic 5nM DA by constructing LP I_h_ G_max_ activity-dependence curves (Fig. 3). The experimental logic for obtaining activity-dependence curves is shown in Fig. 3A. A recurrent voltage step was used to mimic LP slow wave oscillations. Frequency, duration, and amplitude of the recurrent steps were chosen for each preparation individually depending upon measured activity at t = -10min. For example, to mimic slow wave activity exactly (0% change in duty cycle) the frequency and duration of the recurrent step corresponded to average cycle frequency and LP burst duration at t = -10min, respectively; the step and holding potentials corresponded to the average peak and nadir of the LP oscillation at t = -10min, respectively. As shown in Fig. 3A, burst duration was altered by increasing or decreasing the length of the depolarizing step, but not its frequency, or the step or holding potentials. LP duty cycle is the fraction of the cycle over which the neuron is active (burst duration divided by cycle period; i.e., b÷a in Fig. 2A); because cycle period was maintained constant in these manipulations, both burst duration and duty cycle were altered to the same extent. In the absence of a recurring voltage step, LP was held at its initial resting membrane potential in TTX (-59mV on average).

The basic experiment is as follows: After dissection and cell identification, the STG was superfused with TTX for 5min to block spike and slow wave activity, and TTX was present throughout the remainder of the experiment. Next, at t = 0, LP I_h_ was measured with somatic two electrode voltage clamp (TEVC). After the first measure of LP I_h_, DA was (Fig. 3B) or was not (Fig. 3C) added to the superfusate and LP I_h_ was re-measured after 10min. The voltage of LP was continuously controlled with TEVC throughout the experiment. In the 10min between measures of LP I_h_, a recurrent step was (-75 to +50) or was not (-100) implemented.

To obtain an activity dependence curve, the basic experiment was repeated on different preparations, but burst duration was incrementally altered as shown in Fig. 3A. The changes ranged from a 100% decrease in burst duration/duty cycle (-100, complete activity blockade in TTX) to a 50% increase in burst duration/duty cycle (+50). A single datum was obtained from each preparation. Previous work suggested that LP I_h_ was modulated by changes in duty cycle, rather than simply burst duration [15], and so duty cycle is represented on the x-axes of the activity-dependence curves. The changes in G_max_ vs. duty cycle were plotted, rather than absolute G_max_ vs. duty cycle, because LP I_h_ G_max_ is known to vary across individuals by > 3-fold, and we thought it was possible that this large variability could obscure a subtler regulation. The plotted data were analyzed with linear regressions. These analyses indicated that in the presence of 5nM DA, LP I_h_ G_max_ varied linearly and bi-directionally according to changes in LP duty cycle. Conversely, in the absence of tonic nM DA, LP I_h_ G_max_ did not change according to activity. In sum, tonic 5nM DA enabled a mechanism wherein bi-directional changes in LP duty cycle modified LP I_h_ G_max_. In other words, tonic 5nM DA changed the capacity of the modulator (duty cycle) to alter LP I_h_ G_max_.

**Fig 4:**
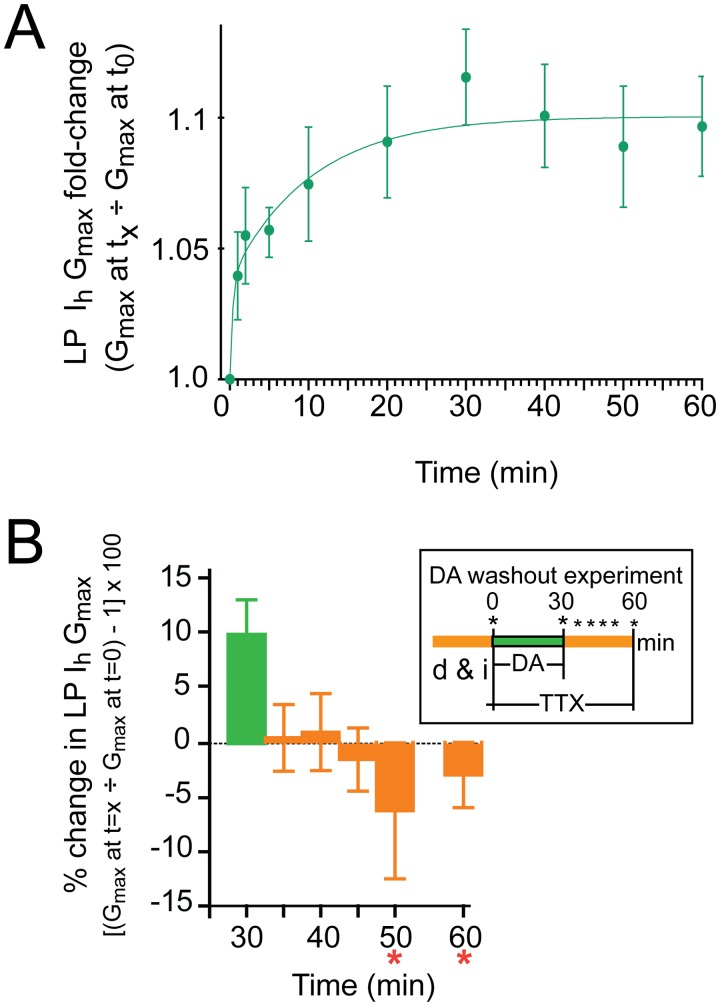
LP I_h_ metamodulation is rapid and reversible. **(A) The time course for metamodulation in 5nM DA**. Experiments measured the fold-change in LP I_h_ G_max_ at 1, 2, 5 and 10min, and every 10min thereafter, up to 60min after addition of 5nM DA. The protocol is as follows: At t = -5min TTX was added to the superfusate bathing the preparation diagramed in Fig. 1A. LP I_h_ was measured at t = 0. The TTX containing superfusate was then supplemented with 5nM DA, and LP I_h_ was re-measured at the indicated time point. One time point was obtained per experiment for 1,2, and 5min. Data for10–60 min represent repeated measures from the same experiment (i.e., in one experiment I_h_ was measured every 10min beginning at t = 10min and ending at t = 60min). Data were plotted as the mean±SEM, n≥4 per time point. The data were best fitted with a double exponential equation yielding time constants of 24sec and 9.9min. **(B) Removing DA rapidly abolished LP I**_**h**_ **metamodulation**. The experiment is diagrammed in the inset; d&i, dissection and cell identification; asterisks indicate measures of LP I_h_ with TEVC. The percent change in LP I_h_ G_max_ relative to t = 0 is plotted (mean±SEM, increases are positive, decreases are negative). Asterisks indicate significantly different from t = 30min as determined using a repeated measures ANOVA with Dunnett’s post-hoc tests that compared all time points to t = 30min, F(5,5) = 2.677, p = 0.0453.

Additional experiments were performed to determine the time course of the DA-enabled change in LP I_h_ at-100 (complete activity blockade with TTX). After dissection and cell identification, the STG was superfused with saline containing TTX for 5min. LP I_h_ was measured with TEVC and 5nM DA was added to the superfusate at t = 0. LP I_h_ was re-measured at various time points thereafter. The mean fold-changes in LP I_h_ G_max_ were plotted against time, and the time constants (Ƭ) for the change in LP I_h_ G_max_ were estimated by fitting an exponential equation to the means (Fig. 4A). The data could not be fitted with a single exponential, but were well fitted with a double exponential, yielding an estimated very fast Ƭ of ~24sec and a fast Ƭ of 9.9min. Note that the very fast Ƭ reflects a best estimate because the first data point was obtained well after 24sec; as such, the rapid process underpinning the very fast Ƭ will not be considered further here, and this study will concentrate only on the mechanism responsible for the fast Ƭ.

Metaplasticity persists beyond the initiating stimulus; metamodulation does not. Next we examined if the change in LP I_h_ G_max_ at-100 persisted upon DA washout. The experiment is diagrammed in the inset of Fig. 4B. After dissection and cell identification, the preparation was superfused with saline containing TTX for 5min. At t = 0, LP I_h_ was measured with TEVC and then 5nM DA was immediately added to the superfusate. At t = 30min LP I_h_ was re-measured and DA was then removed from the superfusate. LP I_h_ was subsequently re-measured at the indicated times during the DA washout. The data suggested that the DA-enabled, activity dependent change was reversible and disappeared within minutes of DA removal (Fig. 4B). Thus, this is an example of metamodulation of LP I_h_ in tonic nM DA.

Metamodulation of LP I_h_ could explain why LP-on phase is stabilized in the presence but not absence of DA during 4AP bath application (Fig. 2). Under our experimental conditions, a 1min bath application of 1mM 4-AP produced a significant 15.13±0.06% increase in LP duty cycle by reducing I_A_ (paired t-test, duty cycle at t = 0 vs. t = 1min, p = 0.0175, n = 19). According to the activity dependence curve in 5nM DA (Fig. 3B), the increase in duty cycle will produce a compensatory decrease in LP I_h_ G_max_. This, in turn, will help to maintain the LP I_A_:I_h_ ratio, which regulates LP-on phase [30]. Since there is no compensatory decrease in LP I_h_ in the absence of DA, LP-on phase is not maintained and instead, the uncompensated decrease in LP I_A_ contributes to a persistent phase advance.

### PKA mediates metamodulation of LP I_h_ in tonic 5nM DA

**Fig 5:**
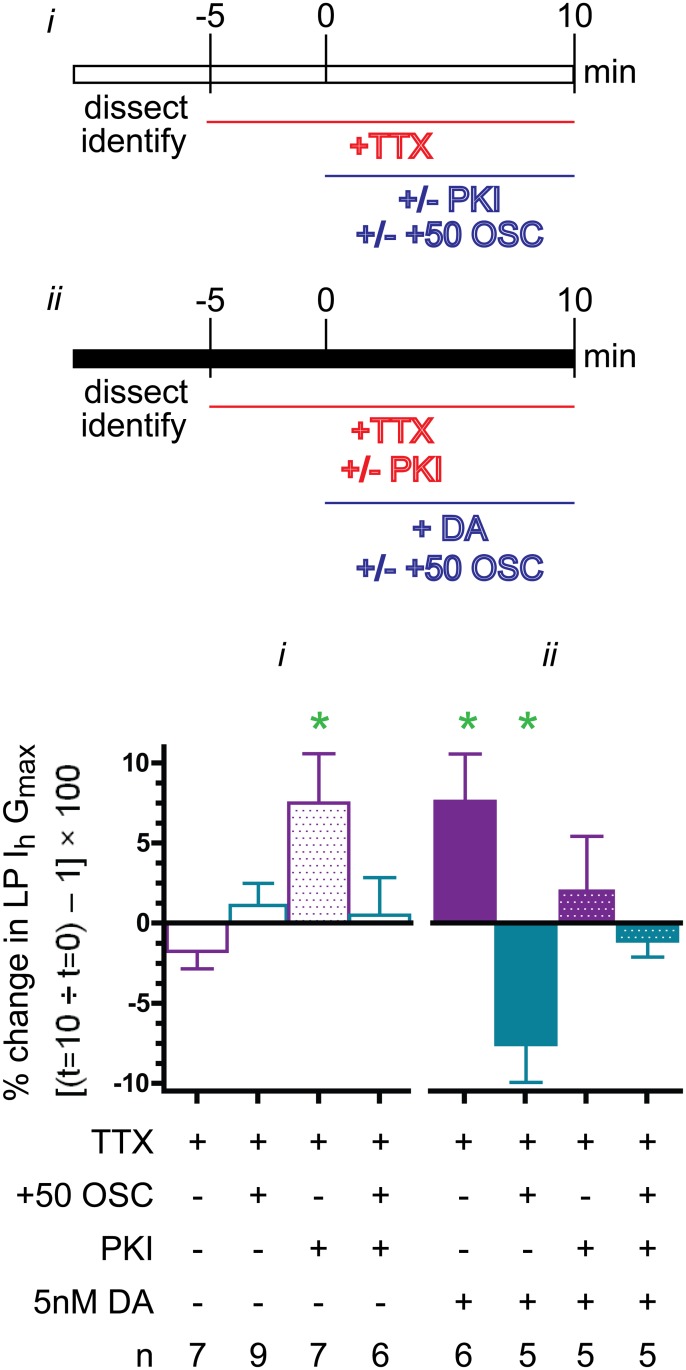
PKA is necessary for metamodulation of LP I_h_ in 5nM DA. Experiments were performed to determine if the specific PKA inhibitor, PKI, blocked metamodulation of LP I_h_ observed at the two extremes of the LP I_h_ activity-dependence curve shown in Fig. 3B; i.e., -100 (TTX alone) or +50 (TTX plus a recurring voltage step mimicking a 50% increase in LP duty cycle, termed +50 OSC). The changes in activity were implemented as previously described for Fig. 3. Two sets of experiments (*i* & *ii*) were performed. The purpose of the first set (*i*) was to test the effect of a 10min PKI application and the second set (*ii*) determined if PKI could block the DA effect. The major differences between the two types of experiments were: 1) PKI was applied at t = 0 in the first set vs. pre-applied at t = -5min in the second set and 2) DA was applied in the second set of experiments. The percent change in LP I_h_ G_max_ at t = 10min relative to t = 0 was plotted (mean+SEM). Paired t-tests compared t = 10min vs. t = 0 within each treatment group; green asterisk, p<0.05. The results show that PKI blocked metamodulation of LP I_h_ in 5nM DA at both-100 and +50.

LP expresses D1Rs. In general, D1Rs can act through the canonical pathway to increase cAMP and activate PKA and/or through any number of other signaling pathways that may or may not involve cAMP [83]. The molecular mechanisms generating metamodulation of LP I_h_ in 5nM DA may change with activity; for example, one set of pathways may be employed at-100 (complete activity blockade in TTX, Fig. 3B) and another may operate at +50 (TTX plus recurring voltage step to mimic a 50% increase in LP duty cycle, Fig. 3B). We next asked if the canonical pathway involving the D1R-PKA axis was necessary for metamodulation in tonic 5nM DA at the two extremes of the activity dependence curve, (i.e. Fig. 3B, -100 and +50). The first series of experiments determined the effect of PKA inhibition on LP I_h_ G_max_ (Fig. 5 panel *i*). The preparation in Fig. 1A was superfused with saline containing TTX for 5min and LP I_h_ was then measured with TEVC at t = 0. Next, the specific PKA inhibitor, PKI, was added to the superfusate, and LP I_h_ was re-measured at t = 10min. From 0 to 10min, LP did (+50 OSC) or did not receive a recurrent voltage step mimicking a 50% increase in LP duty cycle, as described for Fig. 3. The data indicated that global inhibition of PKA significantly increased LP I_h_ G_max_ by 7.5+3.1% when LP activity was blocked but not when LP underwent slow voltage oscillations. This result suggests that in the absence of tonic modulation, PKA influences LP I_h_ G_max_ in an activity-dependent manner. The second series of experiments tested whether DA could induce LP I_h_ metamodulation when PKA activity was inhibited by PKI (Fig. 5 panel *ii*). The preparation shown in Fig. 1A was pretreated with TTX and PKI at t = -5min. LP I_h_ was measured at t = 0 and 5nM DA was subsequently added to the superfusate. LP was superfused for an additional 10min either with (+50 OSC) or without a recurrent voltage step. LP I_h_ was then re-measured. The data showed that preapplication of PKI prevented metamodulation of LP I_h_ under both-100 and +50 conditions; thus, tonic 5nM DA acted through PKA to sculpt the LP I_h_ G_max_ activity-dependence curve. In sum, at-100 PKA activation suppressed or enhanced LP I_h_ G_max_ depending upon the absence or presence of 5nM DA, respectively; whereas at +50, PKA had no effect or decreased LP I_h_ G_max_ in the absence or presence of 5nM DA, respectively. These data suggest that PKA regulation of LP I_h_ G_max_ comprises diverse pathways and that PKA is necessary for LP I_h_ metamodulation.

### Changes in Ca^2+^ concentration underpin activity-dependent alterations in LP I_h_ G_max_

**Fig 6:**
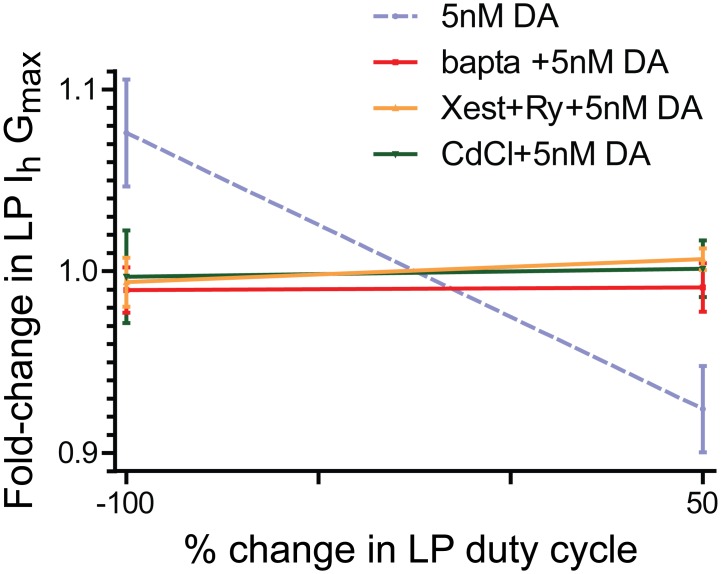
The slope of the LP I_h_ activity-dependence curve in 5nM DA reflects changes in Ca^2+^. Experiments described in Fig. 3B were repeated for-100 and +50, except that an additional drug(s) to disrupt Ca^2+^ dynamics was also continuously superfused beginning at t = -20min (BAPTA or xestospongin C + ryanodine) or t = -5min (CdCl_2_). The fold-changes in LP I_h_ G_max_ (mean+SEM) were plotted for each of the three treatment groups. Linear regression analyses and paired t-tests for each treatment group showed that in every case, the slope of the line was not significantly different from zero and the fold changes at-100 and +50 were not statistically significant. The original experiment from Fig. 3B (dashed line) is shown for comparison.

It is generally accepted that activity-dependent modifications are driven, at least in part, by altered Ca^2+^ dynamics. The purpose of the next set of experiments (n = 5) was to determine if the slope of the activity-dependence curve shown in Fig. 3B reflected changes in Ca^2+^ concentration. The usual experiments to measure LP I_h_ G_max_ at-100 and +50 (Fig. 3A) were repeated for the 5nM DA treatment group except, the preparations were pre-treated with the Ca^2+^ chelator, BAPTA for 20min. A plot of the fold change in LP I_h_ G_max_ at-100 and +50 followed by linear regression analysis showed that changes in Ca^2+^ concentration were necessary to produce activity-dependent alterations in LP I_h_ G_max_ in 5nM DA (Fig. 6). A 20min pre-treatment with BAPTA did not appear to alter the mean LP I_h_ G_max_ at t = 0 (mean±stdev: 0.087±0.003μS, n = 5) relative to untreated controls (0.107+0.028μS, n = 44) (Mann Whitney test, p = 0.062); however, it did significantly alter the variance of I_h_ G_max_ (F test, p = 0.003). Two additional sets of experiments (n = 5 each) were performed to determine if the Ca^2+^ pool being sampled by the DA-enabled mechanism could be influenced by Ca^2+^ entry and/or store release. The usual experiments were performed to measure LP I_h_ G_max_ in 5nM DA at-100 and +50 (Fig. 3A), but either Ca^2+^ entry was blocked with continuous bath application of 0.6mM CdCl_2_ beginning at t = -5min; or, store release was inhibited with continuous bath application of a combination of 10nM ryanodine and 10μM xestospongin C beginning at t = -20min. Note that the latter treatment, which blocks ryanodine and IP_3_ receptors, should result in the emptying of stores. Linear regression analyses of the data indicated that under either of the two conditions, the LP I_h_ G_max_ activity dependence curve had a zero slope. The mean G_max_ at t = 0 for preparations pre-treated with CdCl_2_ (0.105+0.018μS, n = 5) or ryanodine +xestospongin (0.108+0.029μS, n = 5) was not significantly different than untreated control preparations (t-tests, p> 0.05). Together the data show that the activity-dependent mechanism regulating LP I_h_ in the presence of 5nM DA senses changes in Ca^2+^ concentration.

### Tonic 5nM DA simultaneously enables an activity-independent augmentation and a calcineurin-dependent diminution of LP I_h_ G_max_

The opposing actions of kinases and phosphatases may be responsible for activity-dependence, and recent work shows that PKA and the Ca^2+^-dependent phosphatase, calcineurin, are often comprised by the same signalplex to regulate ion current density [84]. In order to determine calcineurin involvement in LP I_h_ metamodulation in tonic 5nM DA, the pharmacological experiments described for Fig. 5 were repeated with calcineurin inhibitors rather than PKI.

**Fig 7:**
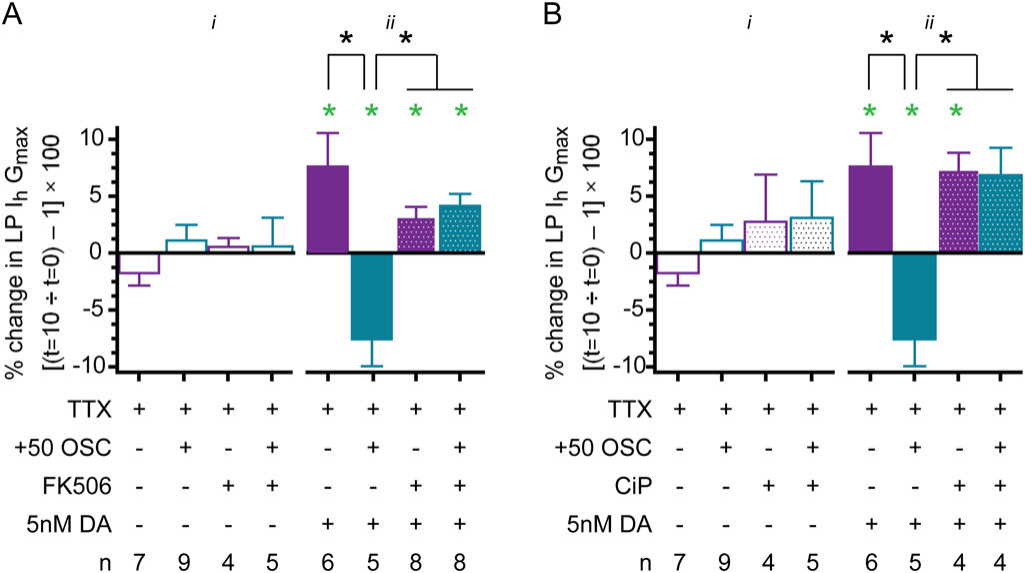
Calcineurin acts as a Ca^2+^ sensor for metamodulation of LP I_h_ in 5nM DA. **(A) The effect of blocking calcineurin with bath applied FK506**. Experiments were performed as described for Fig. 5 using the calcineurin inhibitor, FK506. The percent change in LP I_h_ G_max_ at t = 10min relative to t = 0 (mean+SEM) was plotted. Paired t-tests compared t = 10min vs. t = 0 within each treatment group; green asterisk, p<0.05. Black asterisks indicate that a one-way ANOVA on the four DA-treatment groups with Tukey’s post hoc tests that made all pair wise comparisons showed that the decrease observed for the TTX + DA + (+50 OSC) treatment group was significantly different from the other three DA treatment groups, and that those three DA treatment groups [(TTX+DA, TTX+DA+FK506, TTX+DA+FK506+(+50 OSC)] were not significantly different from one another: (F (3,26) = 11.08; p = 0.0001). There were no significant differences between the 4 treatment groups that did not receive DA, One way ANOVA, F(3,24) = 0.7714, p = 0.5229. **(B) The effect of calcineurin autoinhibitory peptide (CiP) injections**. Experiments in Fig. 5 were repeated except PKI was omitted and CiP was injected into LP at-20min. Paired t-tests compared t = 10min vs. t = 0 within each treatment group; green asterisk, p<0.05. Black asterisks indicate that a one-way ANOVA on the four DA-treatment groups with Tukey’s post hoc tests that made all pair wise comparisons showed that the decrease observed for the TTX + DA + (+50 OSC) treatment group was significantly different from the other three DA treatment groups, and that those three DA treatment groups [(TTX+DA, TTX+DA+CiP, TTX+DA+CiP+(+50 OSC)] were not significantly different from one another: (F (3,18) = 8.71; p = 0.0014). There were no significant differences between the 4 treatment groups that did not receive DA, One way ANOVA, F(3,24) = 1.024, p = 0.4019.

In the first study (Fig. 7A *i*), bath application of FK506 was used to block calcineurin during the usual experiments to measure changes in LP I_h_ after a 10min activity blockade (-100) or a 10min 50% increase in LP duty cycle (+50). DA was not present in this set of experiments. In the absence of DA, bath application of FK506 did not significantly alter LP I_h_ G_max_ at-100 or +50. We interpret the data to mean that in the absence of DA, calcineurin does not influence LP I_h_ G_max_, regardless of LP activity.

Repeating the FK506 experiment in the presence of 5nM DA (Fig. 7A *ii*) indicated that DA permitted calcineurin to regulate LP I_h_ G_max_ in an activity-dependent fashion. We pre-applied FK506 with TTX for 5min and then performed the usual experiments to measure changes in LP I_h_ G_max_ at-100 and +50 in 5nM DA. Blocking calcineurin with FK506 had no significant effect on metamodulation of LP I_h_ at-100; but, it reversed the direction of the change observed at +50. Thus, there was a significant increase in LP I_h_ G_max_ at both-100 and +50 when FK506 was included in the superfusate along with 5nM DA. This augmentation of LP I_h_ G_max_ was not significantly different from that observed at-100 in the presence of DA but absence of FK506; however, it was significantly different from the decrease normally observed at +50 in the presence of DA but absence of FK506. These data indicated that calcineurin regulated LP I_h_ G_max_ in an activity-dependent fashion in the presence of 5nM DA; they suggested that calcineurin functioned as the activity sensor in the machinery mediating metamodulation of LP I_h_. Together with the previous set of experiments, these data indicated that DA enabled the activity sensor. The data also revealed that when the activity sensor was disabled with FK506, 5nM DA increased LP I_h_ G_max_ to the same extent at-100 and +50.

Because FK506 can also alter store release [85], another set of experiments was performed with a second calcineurin inhibitor, calcineurin autoinhibitory peptide (CiP) (Fig. 7B). The usual experiments to measure changes in LP I_h_ at-100 and +50 were repeated in 5nM DA, but instead of bath application of FK506, CiP was injected into LP 20min prior to performing the experiment. The results of blocking calcineurin with CiP injection were comparable to the data obtained from the FK506 experiments: In the absence of calcineurin, 5nM DA did not enable activity-dependent regulation of LP I_h_ G_max_, but instead produced an activity-independent enhancement of LP I_h_ G_max_. In sum, the data indicated that 5nM DA performed two functions: First, it permitted calcineurin to regulate I_h_ G_max_ in an activity-dependent fashion; and second, it increased LP I_h_ G_max_ in an activity-independent fashion.

## Discussion

This study uses a model circuit to examine if/how modulatory tone stabilizes network output over the short-term. The 14-neuron pyloric pattern generator produces a constant rhythmic output. Specific features of the motor pattern are conserved throughout time and across individuals, including the timing of neuronal activity. In all individuals, the Lateral Pyloric neuron (LP) fires a burst of action potentials at the same point in each cycle of the persistent rhythmic motor output. The main finding of the work presented here is that tonic 5nM DA enables activity-dependent regulation of LP I_h_ G_max_ to stabilize the timing of LP activity. This PKA- and calcineurin-dependent mechanism senses the changes in Ca^2+^ concentration that accompany alterations in slow wave activity, which consists of continuous ~1–2Hz voltage oscillations with depolarized plateaus and a ~20mV voltage trajectory ranging from ~-62 to-42mV (Fig. 3). This mechanism results in linear, bi-directional regulation of LP I_h_ G_max_ according to neuronal duty cycle (defined here as the fraction of the cycle period over which LP exhibits a depolarized plateau). In the absence of tonic DA, slow wave activity does not regulate LP I_h_ G_max_, and the DA effect disappears upon DA washout. In sum, tonic nM DA stabilizes neuronal output by enabling activity dependent regulation of LP I_h_ G_max_. This non-persistent effect represents LP I_h_ metamodulation.

### A working model of the mechanism underpinning LP I_h_ metamodulation

**Fig 8:**
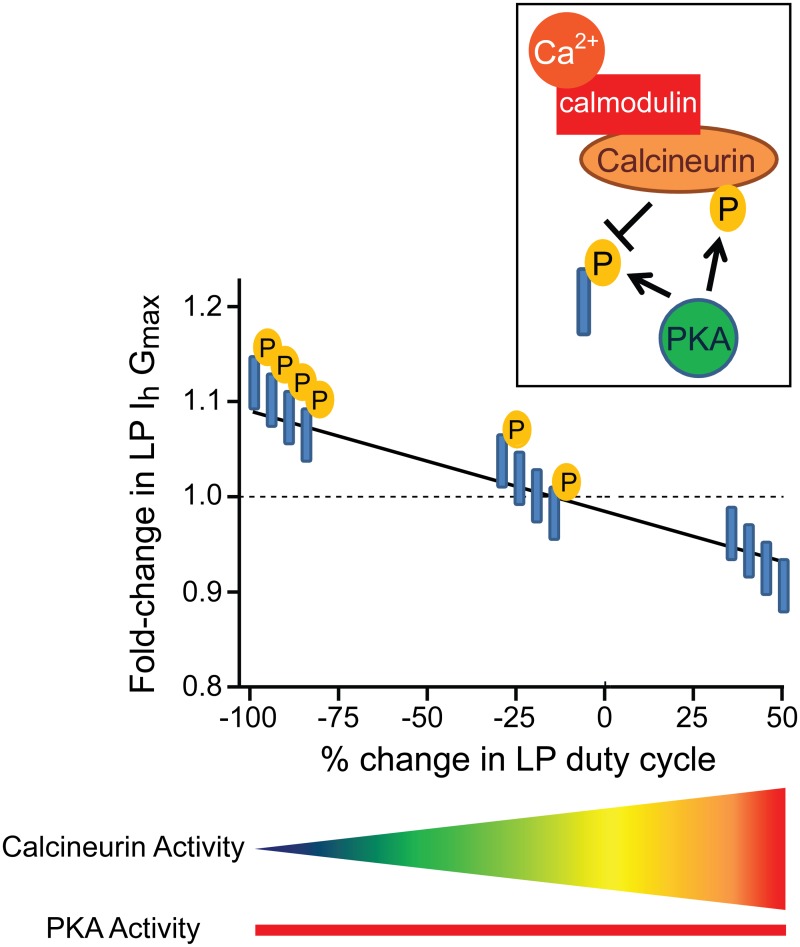
The simplest working model for how tonic nM DA acts through PKA and calcineurin to enable bi-directional, activity-dependent regulation of LP I_h_ G_max_. The graph represents the LP Ih Gmax activity dependence curve in 5nM DA (taken from Fig. 3B). The line indicates the idealized fold change in LP Ih Gmax observed in response to a 10min alteration in LP duty cycle. The boxed inset shows the putative molecular mechanism that is activated by tonic 5nM DA. First, DA acts through LP D1Rs to activate PKA. In turn, PKA phosphorylates an unknown protein (blue cylinder) to increase LP Ih Gmax. PKA also phosphorylates calcineurin, a phosphatase that is regulated by the Ca2+-calmodulin complex. PKA phosphorylation permits calcineurin regulation of LP Ih Gmax. When calcineurin is activated by both PKA and Ca2+-calmodulin, it dephosphorylates the unknown protein to reduce LP Ih Gmax. In the absence of PKA, calcineurin cannot influence LP Ih Gmax. The phosphorylation state of the unknown protein is superimposed upon the LP Ih Gmax activity-dependence curve in 5nM DA. Ca2+ is lowest at-100 and highest at +50, and the gradient below the graph indicates how calcineurin activity changes with LP duty cycle. PKA activity is constant because 5nM DA is tonically present (red bar). The unknown protein is fully phosphorylated at-100 because PKA is active but calcineurin is not. At +50 the unknown protein is completely dephosphorylated because calcineurin activity is maximal. In the idealized state, the unknown protein is partially phosphorylated at baseline (0). This phosphorylation is not DA-dependent. Instead, DA acts to increase baseline phosphorylation of the unknown protein through PKA and to enable activity-dependent regulation of the unknown protein’s phosphorylation state by calcineurin. In the absence of DA, the unknown protein maintains its baseline phosphorylation regardless of LP activity.

Tonic 5nM DA permits changes in slow wave activity to drive bi-directional alterations in LP I_h_ G_max_. In the presence of 5nM DA, a 10min alteration in LP duty cycle produced a graded change in LP I_h_ G_max_. The LP I_h_ G_max_ activity dependence curve is an x-y plot of the percent change in LP duty cycle vs. fold-change in LP I_h_ G_max_. The two extremes of the curve, -100 and +50, represent complete activity blockade in TTX and a 50% increase in LP duty cycle, respectively. In the absence of DA, the slope of the activity dependence curve is zero (Fig. 3C). In the presence of tonic 5nM DA, the activity-dependence curve has a negative slope with the two extremes showing a ~10% increase (-100) or decrease (+50) in LP I_h_ G_max_ (Figs. 3B and 8). The results of our experiments suggest a straightforward mechanism to generate this activity-dependence curve, as diagrammed in Fig. 8.

In our working model, DA acts through PKA to produce two simultaneous effects: A “priming event” that enables calcineurin regulation of LP I_h_ G_max_ and an increase in LP I_h_ G_max_. In the simplest case, the effects are due to direct PKA phosphorylation of calcineurin and an unknown protein (blue cylinder in Fig. 8). Activity-independent PKA phosphorylation of the unknown protein increases LP I_h_ G_max_. Calcineurin opposes the increase in LP I_h_ G_max_ by dephosphorylating the unknown protein. Once the priming event occurs, calcineurin activity is defined by Ca^2+^ concentration, which in turn, is influenced by LP slow wave activity. Based on data showing calcineurin inhibition has no effect at-100 but abolishes the decrease in LP I_h_ G_max_ at +50, the model assumes that-100 represents the lowest Ca^2+^ concentration and the least calcineurin activity. The model also predicts that the unknown protein can be partially phosphorylated at baseline (0, x-axis). The DA-independent mechanism(s) that regulates baseline phosphorylation is unknown, but differences in baseline phosphorylation could account for the variability of individual responses [86]. For example, in some cases an individual showed very little change in LP I_h_ G_max_ in response to a 50% increase in duty cycle or complete activity blockade (examine actual data points at +50 and-100 in Fig. 3B). It may be that baseline phosphorylation varies, and for those individuals showing little response, the unknown protein was almost completely dephosphorylated or phosphorylated under baseline conditions, respectively.

Our data suggest that calcineurin and PKA have opposing actions on LP I_h_ G_max_. The working model depicts this in the simplest way, but it is always possible that calcineurin and PKA do not regulate the same phosphorylation site on the unknown protein, or even the same protein. The unknown protein could be an HCN channel. A-kinase anchoring proteins (AKAPs) are scaffolds that organize multi-protein complexes containing G-protein coupled receptors, ion channels and enzymes [84,87]. The biophysical properties and surface expression of ion channels can be locally regulated by AKAP signalplexes containing PKA and calcineurin [88–93]. When such AKAP signalplexes are anchored to L-type Ca^2+^ channels, they bi-directionally regulate channel phosphorylation state and mean open time [92,94,95]. In this case, the components of the regulatory machinery are highly co-localized with the channel, and it is thought that the mechanism senses local changes in Ca^2+^ concentration resulting from L-type Ca^2+^ channel opening and closing. Regulation of LP I_h_ G_max_ is slow relative to L-type Ca^2+^ channels, with the peak effect occurring ~20 vs. 3min after the initiating stimulus, respectively. This might suggest that the Ca^2+^ pool sensed by the I_h_ mechanism changes more slowly, and/or that the process(es) regulating LP I_h_ G_max_ may be slower; for example, LP I_h_ G_max_ regulation could involve channel recycling rather than gating. In this regard, it is noteworthy that under certain conditions HCN channel surface expression can be regulated over minutes by neuronal activity [96,97].

A single cell contains a mixture of signaling complexes that can generate either local or global signals [98]. Unlike fast and transient phasic modulation, tonic signaling may not be temporally or spatially constrained, and tonic signalplexes could be designed for global regulation. LP D1Rs are located on fine neurites and terminals [47,79] and a study on the Pyloric Dilator neuron demonstrated that DA receptors were only on 40% of the neurites [57]. It will be interesting to determine if the HCN channels being regulated are restricted to the vicinity of the high affinity D1Rs, or neuritic compartments containing D1Rs or if tonic nM DA confers activity-dependence on all HCN channels.

The experimental data suggested that high affinity LP D1Rs acted through PKA to permit calcineurin regulation of LP I_h_ G_max_. In our simplest case model, PKA was depicted as acting directly on calcineurin (Fig. 8), but this was not experimentally demonstrated. PKA priming could also be indirect; for example, PKA could act on a calcineurin regulatory protein or HCN channels to permit calcineurin regulation of I_h_. Alternatively, 5μM DA increases store release in the pyloric Anterior Burster neuron [51], and such a mechanism might also indirectly influence calcineurin activity.

Pharmacological experiments using BAPTA, Cd^2+^, ryanodine and xestospongin C to disrupt normal LP Ca^2+^ dynamics showed that the negative slope of the LP I_h_ G_max_ activity-dependence curve reflected changes in Ca^2+^ concentration; furthermore, both store release and Ca^2+^ entry could influence the pool of Ca^2+^ being sensed. Consistent with these findings, 5μM DA, which modulates Ca^2+^ entry and release [51,65,69], transformed the linear activity dependence curve obtained in 5nM DA into a sigmoid function [15]. Ca^2+^ entry may be through T-type Ca^2+^ channels (CaV3) which are known to be present in crustaceans [99]. A CaV3 window current exists in the voltage range traversed by the slow waves, and this tonic current can be blocked by the high Cd^2+^ concentrations used in our study [100,101]. Entry could also be voltage insensitive, e.g., store operated channels. We previously demonstrated that spike activity may also influence the pool of Ca^2+^ being sensed [15]. In 5μM DA, a 10min 25% reduction in LP duty cycle produced a ~12% reduction in LP I_h_ G_max_. Including spikes on top of the slow voltage oscillations slowed the rate of change in LP I_h_ G_max_ by ~6-fold. We interpreted this finding to mean that spike activity opposed the decrease in average steady-state Ca^2+^ elicited by the change in slow wave activity. It may be that spike activity only affects the apposite Ca^2+^ pool in the presence of μM DA because, the change in I_h_ G_max_ in 5nM DA appeared to follow a similar time course in the presence (Fig. 2) or absence (Fig. 4) of spiking.

Consider that enzymes have different affinities for the Ca^2+^-calmodulin complex (CAM): Calcineurin has a relatively high affinity for CaM, while other proteins, like CAM kinases, have lower affinities [102]. Thus, as the Ca^2+^ concentration rises, calmodulin activity will saturate and the activity of low affinity proteins will increase. This predicts that the metamodulatory mechanism represented by the model (Fig. 8) may only operate within a specific range of Ca^2+^ concentrations and duty cycles. Consistent with this idea, our unpublished preliminary data suggest the linear relationship between LP I_h_ G_max_ and duty cycle may be lost or reset when duty cycle is increased beyond 75%. Perhaps other modulators enable different compensatory mechanisms that function during longer duty cycles; but then again, LP duty cycle is another activity feature that is preserved [29].

### LP I_h_ metamodulation stabilizes the timing of LP activity

Studies on LP I_h_ indicate that DA effects vary according to DA concentration. Our experiments revealed that bath application of 5nM DA+TTX enhanced LP I_h_ G_max_ without changing voltage dependence, whereas a previous study demonstrated that bath application of 100μM DA in TTX decreased the mean LP I_h_ G_max_ by ~20% and shifted the current’s voltage dependence [30]. These data suggest that high and low affinity LP D1Rs have distinct functions.

Our overarching hypothesis is that high concentrations of modulators resulting from phasic release (~μM), act at low affinity receptors and modify circuit output to fit the situation at hand, while low-level tonic modulation (~nM) acts at high affinity receptors to stabilize circuit output by maintaining conductance correlations. Consistent with this idea, here we showed that reducing I_A_ with 4AP increased LP duty cycle and advanced the timing of LP activity phase; however, the timing of LP activity phase was restored in preparations superfused with 5nM DA because, as indicated by the activity-dependence curve (Fig. 8), the increased duty cycle drove a compensatory decrease in LP I_h_. LP duty cycle may be altered by mechanisms that do not involve modifications to Kv4 channels. In these cases, LP I_h_ metamodulation could theoretically distort rather than preserve the I_A_:I_h_ ratio. We think this is unlikely and speculate that future studies may reveal that duty cycle influences the I_A_ window current resulting from Kv4 channel kinetics and voltage dependencies of activation and inactivation; and therefore, under normal circumstances LP I_h_ metamodulation may compensate for dynamic, activity-dependent changes in the tonic I_A_. Alternatively, AKAP signaling complexes are known to influence Kv4 channel trafficking [90,103], and activity may control Kv4 surface expression in the presence and/or absence of DA.

Normally LP acts to slow pyloric cycle frequency by inhibiting the pacemaker kernel through the LP-PD synapse (Fig. 1C) [26]. The timing of LP inhibition during the PD oscillation is critical for this function, and an LP phase advance during an ongoing rhythm can block or even reverse normal LP function, i.e. cause an increase in cycle frequency [27,28]. Given that the timing of LP activity was restored in the 4AP+5nM DA treatment group, one might expect cycle frequency would also be reinstated, but this was not observed. The LP-PD synapse should influence cycle period at the very fast cycle frequencies (≥2Hz) observed in our 4AP experiments [26,28]; however, other neurons and LP conductances can influence cycle period, and 4AP produces alterations in the firing properties of all pyloric neurons [82]. It is likely that multiple modulators regulate numerous conductance correlations in a cell specific manner [21,32,104], and restoration of additional correlations is necessary to restore cycle frequency.

### Multiple homeostatic mechanisms acting over distinct time courses maintain conductance correlations to stabilize neuronal activity features

Every biological cell-type has invariant characteristics that are sustained by feedback mechanisms, and this gives rise to the phenotype that identifies each cell-type. A synthesis of numerous parallel lines of inquiry led to the emergent idea that conductance correlations define neuronal activity features, and feedback mechanisms preserve invariant activity features by maintaining their underlying conductance correlations [105]. Multiple cell-type specific conductance correlations have been observed in the stomatogastric nervous system, and recent studies focus on identifying the correlation(s) underpinning a given activity feature and on modeling the homeostats [21,104,106–110].

The homeostatic mechanisms that operate to maintain conductance correlations and invariant activity features are largely undefined. Studies on the stomatogastric nervous system indicate that these mechanisms are modulator-dependent [15,32,43,111–113]. Some conductance correlations persist when descending modulatory input is removed by cutting the *stn* (Fig. 1), suggesting modulator-independent homeostatic mechanisms may also exist [32,112]; however, other sources of tonic modulation are present under these conditions, including the STG neurons themselves [114] and peripheral inputs [115].

Experimental and computational studies suggest that conductance correlations are underpinned by correlations between their ion channel transcript numbers [106,107,116–118]. Typically, mechanisms that regulate protein expression at the level of the transcript are fairly slow. We previously demonstrated that tonic 5nM DA could enable co-regulation of LP I_A_ and I_h_ through a relatively slow process (~2–3hrs from stimulus to peak change in current) that required microRNA transcription and mTOR-dependent translation [42–45]. This mechanism evoked LP I_A_ plasticity and I_h_ metaplasticity wherein DA once again permitted changes in activity to alter I_h_ G_max_. MicroRNAs are regularly incorporated into feedback loops that control the expression of given proteins; these microRNAs frequently regulate the translation of mRNAs encoding transcription factors, which in turn, influence the expression of these given proteins’ genes [119–121]. Thus, the slow DA-enabled mechanism we described might regulate the translation of a transcription factor(s), which in turn, would control the transcription of Kv4 and HCN channel genes. Since a given microRNA coregulates the translation of multiple transcripts [122], this DA-enabled mechanism might also influence the translation of Kv4 and HCN ion channel transcripts in addition to, or instead of, transcription factor mRNA.

Slow processes to maintain transcript numbers may establish limits for ion channel expression and long-term coordination between ion channels; but, faster mechanisms must also operate to maintain the conductance correlation when one of the correlated currents is rapidly altered. Indeed, LP Kv4 and HCN transcript numbers are known to be positively correlated [32]; however, here we have shown that LP I_h_ G_max_ varies across individuals by >330% when LP Ca^2+^ concentration is not experimentally constrained (I_h_ G_max_ ranges from 0.047–0.156μS), but if Ca^2+^ is clamped to the BAPTA EC_50_ in all LP neurons, then within 20min variability across neurons is reduced to <7% (I_h_ G_max_ ranges from 0.085–0.091μS)! This indicates that HCN transcript number alone does not determine LP I_h_ G_max_; Ca^2+^-dependent post-transcriptional mechanisms are also in play, including the relatively fast metamodulatory mechanism described here (~20min from stimulus to peak change in current). The relatively fast and slow 5nM DA-dependent processes are both mediated by PKA; but, it is not clear if they are sequential or simultaneous and/or can be uncoupled.

We have described two DA-enabled mechanisms for coregulating LP I_A_ and I_h_, but other mechanisms exist as well. Overexpression of Kv4 channels in LP neurons over days in culture produced a compensatory increase in LP I_h_, but unlike DA-enabled metaplasticity, the machinery involved was not activity- or transcription-dependent [14,25]. The authors did not ascertain if modulators enabled this mechanism.

Tonic and phasic DA modulation have distinct functions. It was previously suggested that tonic modulation can set the gain of the response to phasic modulation through processes like receptor desensitization [123]. Our studies show that tonic nM DA can also determine the response to phasic DA by regulating ionic current densities over multiple time scales. If the effects of DA on LP I_h_ can be generalized to other modulators and conductances, then this would suggest that low-level modulatory tone stabilizes neuronal identity by permitting activity-dependent regulation of ion channels to maintain conductance correlations, and this would enable neurons and circuits made from disparate components to similarly respond to a given perturbation that acts on one or more of the disparate components [86,124,125].
